# A Comparative Analysis of the Efficacy, Safety and Mechanism of Action of Flebogamma DIF, Fostamatinib and Romiplostim in Immune Thrombocytopenia

**DOI:** 10.3390/life16030440

**Published:** 2026-03-09

**Authors:** Mary Akinyemi, Kamna Ravi, Furong Tian, Baljit Singh

**Affiliations:** 1 School of Food Science and Environmental Health, College of Sciences and Health, Technological University Dublin (TU Dublin), D07 H6K8 Dublin, Ireland; c21376016@mytudublin.ie; 2 NanoLab Research Centre, Physical to Life Sciences Research Hub, Technological University Dublin (TU Dublin), D08 CKP1 Dublin, Ireland; baljit.singh@tudublin.ie; 3 MiCRA Biodiagnostics Technology Gateway and Health, Engineering & Materials Science (HEMS) Research Hub, Technological University Dublin (TU Dublin), D24 FKT9 Dublin, Ireland; kamna.ravi@tudublin.ie

**Keywords:** immune thrombocytopenia (ITP), platelets, fostamatinib, flebogamma DIF, romiplostim, therapeutic strategies

## Abstract

Immune thrombocytopenia (ITP) is an autoimmune disorder characterized by immune-mediated platelet destruction, resulting in a platelet count below 100 × 10^9^/L and an increased risk of bleeding complications that significantly impair quality of life. Despite advances in ITP management, the unpredictable and heterogeneous nature of the disease continues to challenge treatment selection. This review compares the efficacy, safety, and mechanisms of action of Fostamatinib, Flebogamma DIF, and Romiplostim in adult and pediatric ITP patients. Peer-reviewed studies published over the past 20 years, including randomized and non-randomized clinical trials, observational studies, and real-world evidence, were screened for relevance, with data extracted on dosage, response rates, safety outcomes, and patient characteristics. The sample sizes varied across studies and were reported when available. Study quality and risk of bias were assessed using the ROBINS-I tools. Flebogamma DIF produced rapid increases in platelet count, although its effects were transient. Fostamatinib, the only oral spleen tyrosine kinase inhibitor approved for ITP, was reported to have demonstrated clinical benefit in adults with refractory disease but was contraindicated in pediatric populations. Romiplostim was reported to show sustained platelet responses and facilitate treatment-free remission, particularly in chronic ITP, with an elevated risk of thrombosis. Overall, these therapies offer distinct advantages depending on disease chronicity, patient age, comorbidities, and treatment history. This review underscores the importance of personalized treatment strategies and highlights the need for further long-term, comparative studies to guide evidence-based ITP management.

## 1. Introduction

### 1.1. Overview and Pathophysiology of ITP

Autoimmune diseases constitute conditions in which the immune system loses efficiency and targets healthy tissues, cells and organs, thereby contributing to inflammation and organ malfunction [1,2]. Immune thrombocytopenia (ITP) is an autoimmune disease that is characterized by the production of antiplatelet antibodies. This disease is diagnosed by a platelet count of less than 100,000/mm^3^ when no secondary causes of ITP are evident [3,4]. While ITP is predominantly asymptomatic, some patients may experience bruising, mild bleeding, or, in rare cases, life-threatening hemorrhage. The exact cause remains unclear, though myelodysplastic syndromes, medications, and genetic factors can be considered as probable causes of recurrent ITP [5].

Treatments that can avert bleeding and can sustain platelet count over acceptable levels are crucial for ITP management [6]. Traditional treatments like splenectomies and corticosteroids were primarily used; however, it has been discovered that these treatments can elevate platelet count, plasma cholesterol, leukocyte numbers, and C reactive protein (CRP), and are associated with thrombotic risk, making them unsuitable for long-term use. These limitations highlight the need for safer and more durable therapeutic alternatives [7]. ITP affects both adults and children worldwide, with incidence varying by age and region. Children mainly present with acute ITP, whereas adults are more likely to develop chronic disease [8]. Treatment costs can be substantial, particularly due to outpatient care requirements [9].

Patients frequently report reduced energy, impaired daily functioning, and diminished work capacity, highlighting the impact of ITP on quality of life [10]. ITP involves immune-mediated platelet destruction and impaired megakaryopoiesis [11]. ITP can either be primary, where the exact stimulates are not yet fully understood, or secondary, which has been found to result from external factors such as drugs and other autoimmune disease such as systemic lupus erythematosus [12]. The affected immune system activates the B and T cells that triggers autoantibody formation along with comprised Treg function, which contributes to platelet destruction. The Fcγ receptors on macrophages facilitate platelet degradation; meanwhile, M1 polarization exacerbates the destruction process. Through the Kupffer cells in the liver, the desialized platelets are removed autonomously [6]. ITP is classified as acute (<3 months), persistent (3–12 months), or chronic (>12 months) [13].

### 1.2. Traditional Treatment Approaches and Necessity of Novel Methods for ITP Management

Corticosteroids remain the primary first-line therapy due to their rapid response and low cost [14]. Regardless, long-term use is limited by toxicity, relapse, and steroid resistance, making them suitable only for short-term management [15]. Splenectomy, once widely used, is now reserved for selected patients due to surgical risks and unpredictable outcomes. Many conventional therapies provide only transient benefit, require repeated administration, and fail to maintain long-term platelet stability, negatively affecting quality of life [16]. Second-line treatment options like rituximab have demonstrated an immunosuppressive effect on patients due to B-cell reduction; however, it carries risks of immunosuppression and developing infections [17].

These limitations have driven the development of novel therapies like TPO-Ras, which can induce remission in both acute and chronic ITP patients after about 1–2 years of treatment, thereby eliminating the requirement for lifetime use [18,19]. Syk inhibitors reduce thrombotic risk and are suitable for patients prone to thrombosis [20]. IVIG provides rapid platelet elevation and demonstrates enhanced efficacy when combined with steroids [18]. These therapies offer contrasting benefits and limitations in increasing the platelet count with their long-term safety and efficacy subject to ongoing research.

### 1.3. Mechanism of Action Associated with ITP

**Figure 1:**
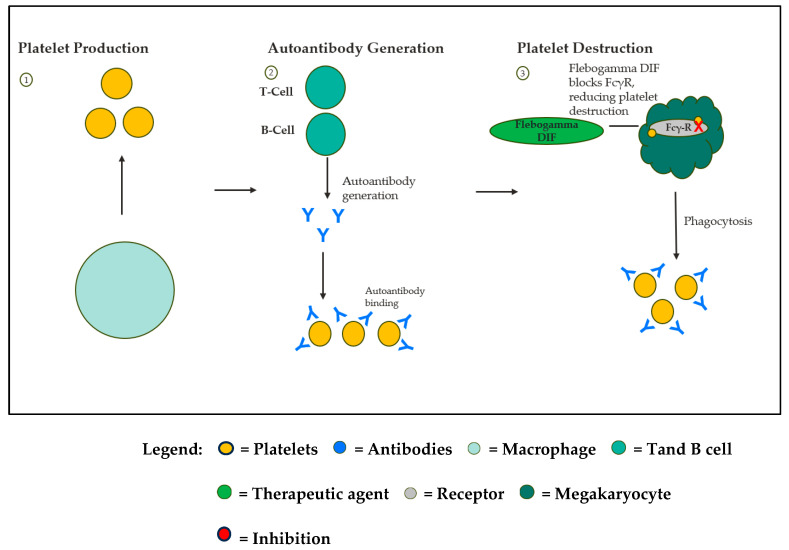
Operation of Flebogamma DIF in ITP. Flebogamma DIF (IVIG) functions by interfering with platelet destruction by macrophages by blocking the Fc receptors on macrophages and modulating B- and T-cell activity. ITP, immune thrombocytopenia; IVIG, intravenous immunoglobulin. Created in Microsoft PowerPoint.

**Figure 2:**
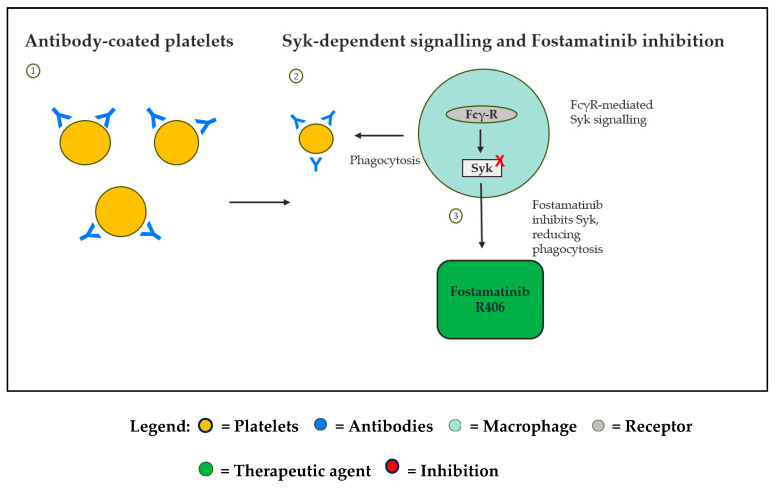
Fostamatinib (Syk inhibitor) blocks the Fc-receptor-mediated activation of macrophages, allowing it to have a targeted action on the macrophage activity and reducing the phagocytosis of antibody-coated platelets, which aids in preventing platelet destruction in ITP. Syk, spleen tyrosine kinase; Fc-R, Fc receptor; ITP, immune thrombocytopenia. Created in Microsoft PowerPoint.

Flebogamma DIF (IVIG), Fostamatinib, and Romiplostim tackle different elements of ITP pathophysiology (Figure 1). Flebogamma DIF modulates Fcγ receptors on macrophages, reducing immune-mediated platelet destruction [21]. In Figure 2, Fostamatinib inhibits Syk-mediated Fc-receptor signaling, decreasing macrophage phagocytosis of antibody-coated platelets [22]. Syk is a crucial effector of the Fc-receptor and B-cell receptor signaling in inflammatory cells such as macrophages [23].

**Figure 3:**
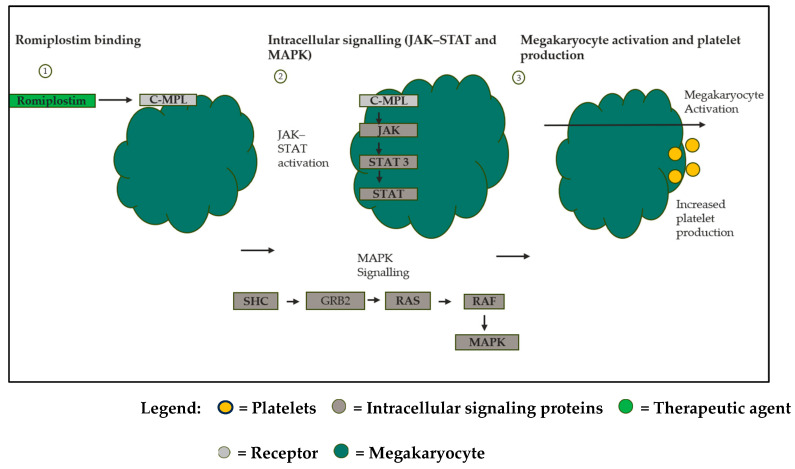
Operation of Romiplostim in increasing the platelet count in ITP. Romiplostim (TPO-RA) binds to and activates the thrombopoietin receptor on megakaryocytes and their precursors. This activation leads to the stimulation of the JAK-STAT and MAPK pathway, promoting megakaryocyte proliferation and differentiation, which increases platelet production in patients with ITP. Created in Microsoft PowerPoint.

Romiplostim stimulates megakaryocyte proliferation and platelet production via TPO-receptor activation (Figure 3) [24]. Combination approaches have been explored, but evidence remains insufficient to determine their potential benefit. IVIG with Romiplostim has been reported to have the ability to rapidly elevate platelet counts in refractory ITP [25]. Given the distinctions in mechanisms of Fostamatinib and Romiplostim, combining these two therapies could potentially show promising responses in patients unresponsive to monotherapies [25]. However, combination therapy could face issues like elevated costs and toxicity [26].

### 1.4. Efficacy of Current Treatment Options

The efficacy of any drug is measured by its capacity to achieve the maximum intended therapeutic effect under ideal conditions [27]. Flebogamma DIF has been reported to provide the fastest platelet response within 24–48 h in approximately 80% of patients [28], supporting its use in situations requiring rapid platelet elevation. However, its effects are transient, lasting for an average of 3–4 weeks, which may limit its suitability for long-term management in chronic ITP. In several studies, Romiplostim has demonstrated high response rates and sustained platelet stability, particularly in chronic ITP, with a median response time of 1–2 weeks [29]. Studies report sustained platelet responses and reduced bleeding episodes with Romiplostim, and a proportion of patients were able to discontinue other ITP therapies [30]. These findings vary across studies and should be interpreted cautiously due to the absence of head-to-head comparisons. Although currently recommended after failure of prior treatments, ongoing research is evaluating its potential as a first-line therapy [31].

Fostamatinib only achieves a platelet response rate in about 40% of patients [32], with a median time to response of around 15 days and a durable response rate of 17% [33]. While Fostamatinib provides greater platelet durability than Flebogamma DIF, this sustained response rate appears lower than rates reported for Romiplostim (61%); however, these comparisons are indirect and should be interpreted cautiously [34]. Romiplostim was also reported to show higher sustained response rates in newly diagnosed ITP (50%) compared to persistent (35.3%) and chronic ITP (31.1%) [35]. Further research is needed to clarify its role in earlier treatment lines [12].

### 1.5. Safety Profile of Therapies and Adverse Events

Based on the differences in the mechanism of action, the safety profiles of each therapy differ. Flebogamma DIF is administered via intravenous (IV) infusion over 2–5 days and is generally well tolerated but may cause infusion-related reactions and temporary adverse events. Serious complications such as thrombosis or renal dysfunction can occur on rare occasions [19]. Fostamatinib is administered orally twice daily, which may offer convenience for some patients. Comparably, Fostamatinib has mild-to-moderate adverse events such as diarrhea, hypertension, and cephalea and is linked to serious adverse effects (SAEs) like sepsis, epistaxis, and liver elevations, limiting its use in treatment of gastrointestinal issues [20,36]. Romiplostim, administered subcutaneously once per week, may support increased adherence in some patients, particularly for pediatric patients. Others, however, prefer oral medications over SC injections due to ease of administration, convivence and reduced time consumption, especially in cases where self-administration of Romiplostim is not possible [37]. SC injections are known for their greater bioavailability and rapid-onset action and are deemed an ideal route of administration from a pharmacokinetic perspective; however, other factors such as patient compliance must be considered [38]. Romiplostim has an acceptable safety profile but carries risks of thrombosis, rebound thrombocytopenia, and bone marrow fibrosis, while common side effects include headaches, fatigue, and injection site infections [39,40].

### 1.6. Comparison and Main Implications of ITP Therapies

While many other ITP therapies such as eltrombopag, avatrombopag, and rituximab are widely used, they were not included in this review because the aim was to compare three mechanistically distinct treatments (IVIG, Syk inhibition, and TPO-RA). This focused comparison allows clearer evaluation of contrasting mechanisms, safety profiles, and clinical roles.

Oral therapies may offer convenience and improved adherence for some treatment plans but may be unsuitable for pediatric patients due to safety concerns, as seen with Fostamatinib [41]. Oral therapies undergo first-pass metabolism in the liver; hence, higher doses may be required to achieve therapeutic levels compared with IV or SC therapies, which bypass this process and therefore provide more predictable pharmacokinetics [42]. Romiplostim and Flebogamma DIF have been approved by the FDA for chronic ITP management in pediatric patients, and since they bypass the first-pass metabolism, they have been reported to be appropriate for patients unable to tolerate oral medications [43]. Nonetheless, parenteral administration has drawbacks such as increased pain, discomfort, infection, etc., and may not be suitable for all patients [44].

While traditional therapies have been reported to be effective, their toxicity and transient effects have led to the need for new therapies such as Flebogamma DIF, Fostamatinib, and Romiplostim, which provide alternative mechanisms of action and may offer improved outcomes in some patients [45,46]. These therapies provide targeted mechanisms that expand treatment options for patients with ITP. However, the question lies as to whether these targeted therapies are a promising alternative globally in terms of cost, availability, etc. This review will critically explore their mechanisms, safety, tolerability, effectiveness, and global approval to support informed treatment decisions.

## 2. Methods and Procedures: Data Analysis

### 2.1. Study Design and Comparison Approach

This study is a structured, narrative review that analyzed data from published studies, including observational research, real-world based studies, and clinical trials, to assess the efficacy, safety, reliability of Flebogamma DIF, Fostamatinib and Romiplostim in adult and pediatric ITP patients. A comparative analysis approach was applied across different relevant literature sources to identify the differences and similarities in treatment outcomes across therapies with distinct mechanisms of action. Data collection and reporting were informed by key principles of the PRISMA 2020 guidelines, and a PRISMA flow diagram is provided to enhance transparency.

Based on the heterogeneity of included studies, including randomized controlled trials, non-randomized trials and observational studies, no formal quantitative synthesis was conducted. In addition to this, baseline differences such as ITP classification, patient age and sex, prior treatment, comorbidities, and sample size were not statistically considered. Instead, a narrative synthesis was carried out to qualitatively evaluate trends in the safety and efficacy of each treatment. Therefore, all comparative interpretations in this review should be considered exploratory and interpreted with caution due to the heterogeneity of included studies. The literature search covered the period from 2005 to 2025.

### 2.2. PRISMA

**Figure 4:**
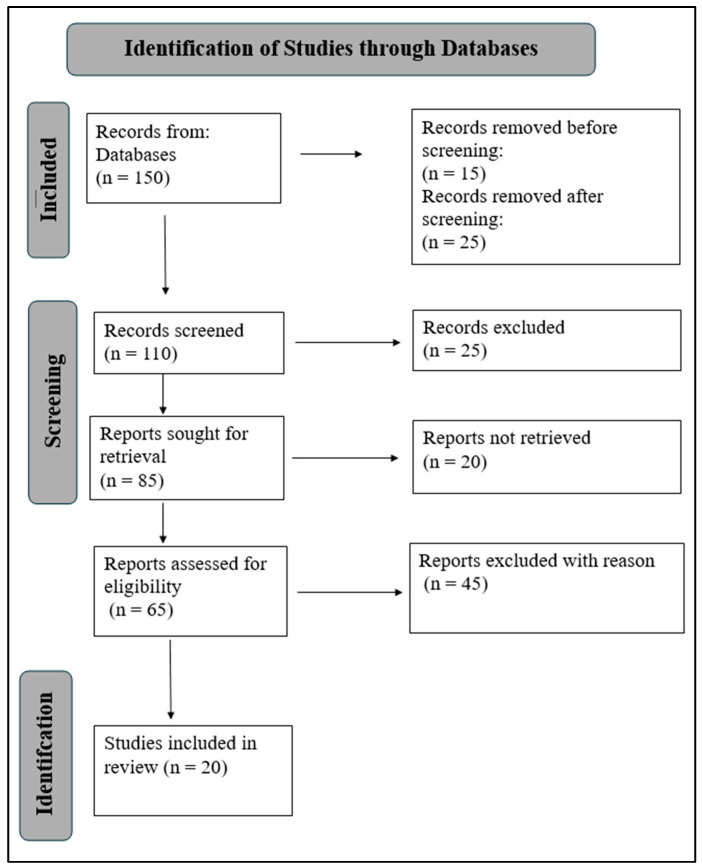
Flow diagram highlighting the application of the PRISMA flowchart in analyzing literature, illustrating the number of articles used in the selection, inclusion, and exclusion process. Prospero Registration Number: CRD420251089681.

The PRISMA 2020 statement was primarily established for systematic reviews of studies that assess the impacts of health interventions, regardless of the design of the studies incorporated [47]. PRISMA includes a flow diagram and a 27-item checklist to aid in the transparency, reliability, and reproducibility of the reviews [48]. Figure 4 illustrates the application of the PRISMA flow diagram for this systematic review, which guided the data inclusion and exclusion process. Figure 4 is the flow diagram that details the number of records identified, screened, excluded, and included in the study. The diagram provides transparency for the number of articles used in the selection, inclusion, and exclusion process. The protocol for this review was registered with PROSPERO (CRD420251089681).

### 2.3. Review Type and Methodological Transparency

Although this review followed PRISMA principles and was prospectively registered in PROSPERO, the methodological approach is best characterized as a systematic narrative review rather than a full systematic review. A quantitative synthesis was not feasible due to substantial heterogeneity in study designs, populations, outcome definitions, and reporting quality. Therefore, the evidence was summarized narratively, and all comparative interpretations should be considered exploratory.

All search results were imported into a reference manager, which automatically detected duplicate records. These were manually checked for accuracy and removed prior to screening. Two independent reviewers screened titles and abstracts, followed by full-text assessment. Any disagreements or uncertainties were resolved through discussion, and a third reviewer reviewed the data and was consulted when necessary.

These procedures were implemented to minimize bias and improve reproducibility within the constraints of a narrative synthesis.

### 2.4. Inclusion and Exclusion Criteria

In accordance with the PRISMA guidelines, a screening process was applied to determine the inclusion and exclusion criteria, which are as follows:

Inclusion criteria: From all the treatments available on the market for ITP, Flebogamma DIF, Fostamatinib and Romiplostim were selected based on their contrasting mechanisms of action and route of administration. Flebogamma DIF is an IVIG that is administered via IV infusions; Fostamatinib is a Syk inhibitor, administered orally; and Romiplostim is a TPO-RA administered via SC injections. These treatments target different areas, enabling better comparative analysis. These three therapies were selected as they represent distinct therapeutic classes (IVIG, Syk inhibition, and TPO-RA), have broad regulatory approval (FDA/EMA), and are widely used across multiple regions. Other approved ITP treatments such as eltrombopag, avatrombopag, and rituximab were not included as they fall outside the defined scope of this comparative review.

Included publications: (1) treatments approved by relevant health authorities such as the FDA and EMA; (2) treatments that were utilized in more than one region globally; (3) publications that detailed the safety, efficacy, dosage, response duration and outcomes; (4) peer-reviewed clinical trials, observational studies, and real-world studies; and (5) publications published in English during the 20-year period from 2005 to 2025 to ensure that data is not outdated and is still available on the market. Where reported, individual study sample sizes are presented in Table 1, Table 2 and Table 3; however, due to variability in reporting, an exact total number of patients across all studies could not be calculated.

Exclusion criteria: Studies were excluded based on the following: (1) non-ITP conditions, e.g., treatments for other autoimmune diseases; (2) articles lacking sufficient data on dosages, sample size, etc.; (3) language limitations, such as those published in non-English or without full-text access; (4) insufficient and unclear information on the safety, effectiveness, sample sizes and outcomes of the treatments; (5) studies involving pregnant or lactating women; and (6) duplicate cohorts or data with no advanced revelations.

### 2.5. Research Process for Screening Study Findings

To determine the treatments utilized for ITP, relevant publications were discovered using the following databases: Google scholar; TU Dublin library; Science Direct; PubMed; American Society of Hematology; Reports from Regulatory agencies and ClinicalTrials.gov; Regulatory databases, e.g., FDA, EMA. These data banks were applied to determine the patient safety, sustainability, and tolerability on the chosen therapies. Database search filters and keywords (including ‘ITP’, ‘Adults’, ‘Pediatrics’, ‘Flebogamma DIF’, ‘Fostamatinib’, ‘Romiplostim’, ‘Tavailsse’, ‘Treatment’, ‘Current’ and ‘Global’) were applied to establish a more precise search. To offer a comprehensive analysis of Flebogamma DIF, Fostamatinib and Romiplostim, essential information from 20 key articles meeting the inclusion criteria was used (refer to Table 1, Table 2, Table 3 and Table 4). These tables consist of articles in which Flebogamma DIF, Fostamatinib and Romiplostim have been demonstrated to be effective at managing the platelet levels in ITP. Study selection, data extraction, and interpretation were performed independently using predefined criteria to minimize potential bias.

### 2.6. Data Extraction and Synthesis

Table 1, Table 2 and Table 3 display 20 articles, with each article highlighting the following: treatment type; quantity of dosage used; patient type (adult and/or ‘pediatrics’); number of cohorts used in the trial; description of the trial; safety and efficacy outcomes; country/region the trial was performed in; study design and reference to the article. The first 5 articles are related to the Flebogamma DIF therapy at a concentration of 5% and 10%. This data is related to pediatric and adult ITP patients with dosages ranging from 0.4 g/kg to 2 g/kg. Fostamatinib is associated with the next 7 articles, with concentrations ranging from 100 mg to 175 mg. Since this treatment is not approved in children under 18 years, this data is focused only on adult patients with ITP. The last 8 articles represent Romiplostim, which is based on adult and pediatric ITP patients with doses ranging from 0.1 µg/kg to 10 µg/kg.

An additional table also illustrates the benefits and limitations to be considered for each therapy based on patient specific factors such as disease severity, length of disease, underlying health conditions, etc. Treatment outcomes were differentiated based on the international ITP classification guidelines—acute, persistent and chronic ITP—wherever available. However, many of the included studies did not clearly state ITP duration or classification inpatients. Similarly, none of the studies distinguish between immune and non-immune ITP. This review focuses primarily on immune ITP, consistent with majority of the literature available.

### 2.7. Quality of Study and Bias Evaluation

The quality of publications used was evaluated based on the study design, sample size, risk of bias and transparency of therapeutic outcomes. As all included studies were non-randomized, the Risk of Bias in Non-Randomized Studies of Interventions (ROBINS-I) tool was applied to assess potential confounding, participant selection, outcome measurement, and reporting bias (see Supplementary Material Tables S1–S3). The application of this tool provides a comprehensive assessment of quality of the study and potential bias that may exist across heterogeneous study designs.

While the study quality and risk of bias were evaluated, these findings were not weighed based on these assessments. Therefore, the narrative synthesis included studies with varying methodological rigor that were interpreted similarly, which may introduce confirmation bias. This limitation, combined with the absence of quantitative synthesis and direct head-to-head trials, restricts the strength of comparative conclusions.

Overall, most studies demonstrated moderate to serious risk of bias, mainly due to confounding, small sample sizes, and incomplete reporting. These assessments informed the interpretation of individual study findings, with higher-quality evidence given greater interpretive weight. Given the substantial heterogeneity across study designs and the absence of head-to-head trials, all cross-study comparisons should be considered exploratory rather than definitive.

### 2.8. Outcome Definitions

In this study, key therapeutic outcomes were defined to ensure consistency across studies used. This is as follows:

- Platelet response is defined as achieving a platelet response ≥ 50 × 10^9^/L following treatment.
- Sustained platelet response is defined as a platelet count ≥ 50 × 10^9^/L maintained over a period of at least 6 months without the need of rescue therapy.
- Baseline platelet level refers to the initial platelet count level recorded prior to initiating treatment.
- Acute ITP is thrombocytopenia that persists for less than 3 months from diagnosis.
- Persistent ITP is thrombocytopenia that persists for 3–12 months from diagnosis.
- Chronic ITP is thrombocytopenia that persists for more than 12 months from diagnosis.
- Mean time to response is referred to as the average time from the initiation of treatment until a clinically significant platelet count increase was observed.
- Adverse events (AE) are defined as any unpleasant reaction in a patient whether it is treatment-related or not.
- Serious adverse events (SAE) refer to adverse events that can be fatal or life-threatening, require hospitalization or result in significant disabilities.

### 2.9. International Market Assessment for ITP Therapies

Flebogamma DIF, Fostamatinib and Romiplostim are approved and available in various regions and countries globally for ITP treatment. All three therapies are approved by major regulatory agencies such as the Food and Drug Administration (FDA) and European Medicines Agency (EMA), with only Romiplostim approved and evaluated for trials in China, specifically only in Chinese adults suffering from persistent or chronic ITP. For the global application of each treatment, refer to Section 3.4.

## 3. Results

All comparisons presented in this review are indirect, as no head-to-head clinical trials between Flebogamma DIF, Fostamatinib, and Romiplostim were identified. The included studies differed substantially in design, patient populations, baseline platelet counts, prior treatments, and outcome definitions. These factors were not adjusted for in this narrative synthesis. Therefore, observed differences in efficacy and safety may reflect study-level heterogeneity rather than true differences in therapeutic performance, and comparative interpretations should be considered exploratory.

### 3.1. Research Articles for the ITP Therapies

**Figure 5:**
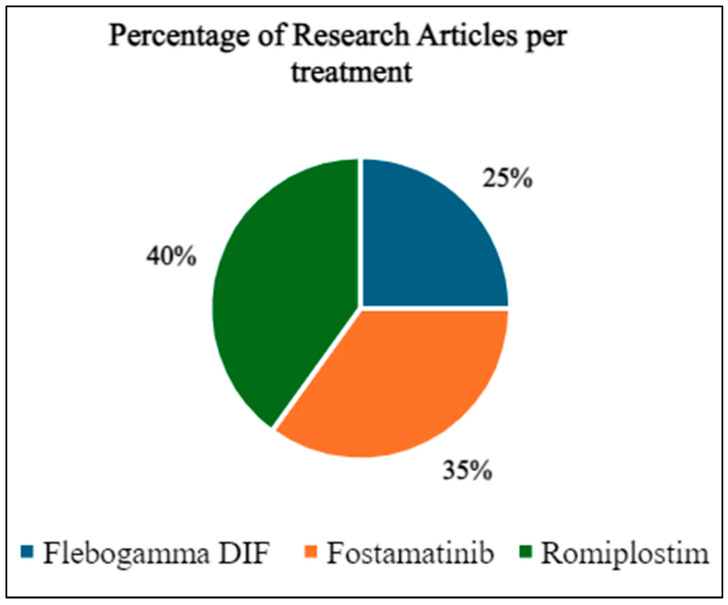
The number of research articles applied for Flebogamma DIF, Fostamatinib and Romiplostim.

The percentage of publications relating to each ITP treatment is seen below in Figure 5. From the literature included, 25% of studies evaluated Flebogamma DIF, 35% evaluated Fostamatinib, and 45% evaluated Romiplostim. Study designs ranged from randomized controlled trials to observational cohorts, with sample sizes varying widely across treatments. Table 1, Table 2 and Table 3 summarize the main findings for each therapy.

### 3.2. Global Approval Status for ITP Therapies

Flebogamma DIF (5% and 10%) received regulatory approval between 2007 and 2010 across North America, Europe, Australia, India, and Colombia. Fostamatinib was approved by the FDA in 2018 for chronic ITP and subsequently authorized in Europe, Canada, Israel, and Japan. Romiplostim was approved in 2008 for adults and in 2018 for pediatric patients, with current authorization in more than 69 countries [49].

### 3.3. Key Findings and Considerations: Advantages and Disadvantages of Key ITP Therapies

The studies reviewed provide insight into the efficacy, safety, and clinical roles of the three ITP therapies. Various studies reported that Flebogamma DIF consistently demonstrated rapid increases in platelet count, supporting its use in acute situations where immediate elevation is required. Studies also report Fostamatinib showing moderate overall responses, with higher effectiveness when used as a second-line therapy rather than in later treatment stages. Romiplostim was associated in various studies with more bleeding events than the other therapies but demonstrated the strongest ability to sustain platelet counts ≥50 × 10^9^/L, particularly in chronic ITP. Across all treatments, most AEs were mild to moderate, indicating that these therapies are generally well tolerated. These findings from various relevant studies highlight the importance of individualized treatment selection based on clinical urgency, prior therapy, comorbidities, and patient-specific risk factors.

Table 1 summarizes the detailed study findings, including efficacy outcomes and safety profiles, for Flebogamma DIF. Table 2 summarizes the detailed findings including efficiency outcomes and safety profiles for Fostamatinib.

**Table 1:** Articles reviewed on Flebogamma DIF: main findings.

		Result	Ref.
**Flebogamma DIF 5%/10%** **(Infusions are administered intravenously over 2–5 consecutive days)**	1	81.3% achieved a platelet count. Mean time—1.7 days. 92.2% revealed mild-to-moderate AEs.	[50]
	2	Platelet count response: 72.2% in trial A (adults) and 81% in trial B (adults + children). Time to reaction—2 days (adults) & 1.5 days (children). TEAEs 38.9% (Trial A) and 30.4/83.3% (adults/children; Trial B). 90.8% revealed AEs.	[19]
	3	TEAEs—18.5% (10% product) & 2.2% (5% product). 17 SAEs with 3 allergic reactions. 46.2% children & 11.3% of adults suffered from treatment associated with AEs.	[51]
	4	PRR of 74% within 2.5 days. 40% displayed iatrogenic AEs.	[52]
	5	71.4% reached target platelet count. 40 non-serious AEs associated with the treatment.	[53]

Footnote: Sample size range: 23–76 patients; dosage range: 0.4 g/kg to 1 g/kg; countries: USA, India, Colombia, Spain, Russia, UK, Canada, Germany.

**Table 2:** Articles reviewed on Fostamatinib: main findings.

		Result	Ref.
**Fostamatinib** **(orally Administered)** **Sample size range: 2–157 patients** **Dosage range: 100 mg twice daily, increased to 150 mg twice daily if inadequate response after 4 weeks** **Countries: North America, Australia, Europe, Japan, Spain, United States**	1	18% attained a stable response (SR) without rescue medications. Overall response rate (ORR) 43% in the first 12 weeks. 78% maintained the response at 12 months and 56% at 24 months.	[54]
	2	PRR—36%. Out of 14 patients, 43% lessened/ceased glucocorticoids without relapse. AE—96%. TEAE—77%	[55]
	3	ORR—85.9% + CR—70.3%. CR—2nd-line treatment (66.6%), 3rd-line (72.1%). Median time to response—63 days. 45.3% had mild-to-moderate AEs & 23.4% discontinued treatment.	[56]
	4	Median duration of treatment was 2 months. 69.5% responded + 45.7% attaining a CR. 45.6% had grade 1–2 AEs & 54.3% discontinued treatment, with 3 fatalities due to severe bleeding.	[20]
	5	PRR—78% (2nd-line therapy) & 47% (3rd-line therapy or later-line treatment). 90% of patients with persistent ITP reacted positively to treatment than those with chronic ITP. ORR in phase 3 was 54%.	[57]
	6	64% achieved a response. Discontinuation of treatment: 10 patients (no response), 3 patients (absence of response) & 1 patient (personal circumstances). Mild-to-moderate AEs.	[58]
	7	Patient 1—Limited need of rescue therapies. Hypertension worsened. Patient 2—Escalation of pre-known IBS necessitating anti-diarrheal therapies.	[59]

Footnote: Sample size range: 2–157 patients; dosage range: 100 mg twice daily, increased to 150 mg twice daily after 4 weeks if inadequate response; countries: North America, Australia, Europe, Japan, Spain, United States.

Table 3 summarizes the detailed study findings including efficacy outcomes and safety profiles for Romiplostim.

**Table 3:** Articles reviewed on Romiplostim: main findings.

		Result	Ref.
**Romiplostim** **(Administered subcutaneously (SC) once weekly)** **Sample size range: 15–1879 patients** **Dosage range: 0.1 μg/kg to a maximum of 10 μg/kg** **Countries:** **Japan, China, Scandinavian countries,** **Europe,** **Canada, Brazil,** **Israel, Turkey, India**	1	Discontinuation due to limited efficacy, AEs & ADRs. After discontinuation: 4.43% (repeated thrombocytopenia) + 6.07% (hemorrhage). 82.93% of severe hemorrhage. 2.22% of ADRs led to death. Thromboembolic-related ADRs in 3.08%.	[60]
	2	Platelet count after discontinuation—100%. TEAE—82.8%. 2 deaths in romiplostim group.	[61]
	3	28% (newly diagnosed), 16% (persistent) & 55% (chronic) ITP. Durable platelet response from wks. 14–52: 64.6%, 52.9% & 52.7%, respectively. Thrombocytosis—29.7%. Thrombotic/thromboembolic episodes—10.2%. Bleeding—21.1%. Cessation in 54.9%. 16.2% required rescue medication.	[62]
	4	80% displayed an increased PLT. 51–74% of patients discontinued all other ITP treatments after starting romiplostim. ADRs occurred in 11–17%.	[63]
	5	Platelet response—89.4%. TEAE bleeding occurred in 68.4%. 7% were in remission after treatment cessation. 25% reported SAEs, 2% had neutralizing antibodies.	[64]
	6	On treatment completion: 67% were in remission, 46% were in steroid-free remission (SROT) excluding those with chronic ITP.	[65]
	7	Median treatment time—155.9 wks. 88.2%—platelet response. 80.1%—platelet count increase. Higher response in home administration vs. clinical administration. 5.4% attained a durable response. AEs in 95.1% + SAEs in 29.6% with only 3.9% associated with the treatment.	[66]
	8	Platelet increase in 42.5% in wk. 1. Durable response—65%. 20% experienced grade 1 & 2 bleeding episodes. Mild-to moderate AE (40.6%).	[67]

Footnote: Sample size range: 15–1879 patients; dosage range: 0.1 μg/kg to 10 μg/kg; countries: Japan, China, Scandinavian countries, Europe, Canada, Brazil, Israel, Turkey, India.

Table 4 summarizes the advantages and disadvantages of Flebogamma DIF (5% and 10%), Fostamatinib and Romiplostim: key considerations.

**Table 4:** Advantages and disadvantages of Flebogamma DIF (5% and 10%), Fostamatinib and Romiplostim: key considerations.

Treatment		Advantages	Disadvantages
**Flebogamma DIF 5% & 10%**	1	Amplifies platelet count to hemostatic levels.	Costly & limited availability.
	2	Inhibits the destruction of IgG-sensitized platelets by the reticuloendothelial system.	Time-consuming.
	3	Safe and effective for adult and pediatric patients (≥2 years)	Possibility of infusion-associated reactions.
	4	Flebogamma DIF 10% has half the volume of the 5% solution, reducing the infusion time & enhancing patients’ lives.	Not suitable for people with hereditary fructose intolerance.
	5	Allows for rapid platelet count increase.	Likelihood of acute renal failure, critical instant hypersensitivity reactions and thromboembolic difficulties.
**Tavalisse (Fostamatinib)**	1	Ease of administration + convenient.	Risk of diarrhea, neutropenia, liver enzyme elevation and hypertension.
	2	Effective + biocompatible in heavily pre-treated ITP patients.	Limited real-world clinical data.
	3	Favorable alternative for patients resistant to eltrombopag or romiplostim.	Not suitable for children under 18 years old.
	4	Less risk of thrombosis compared to TPO-RA	Reduced stable response when applied as a 3rd-line therapy or later.
	5	Decreases antibody-modulated destruction.	Risk gastrointestinal effects.
**Romiplostim**	1	Increases platelet count rapidly & can maintain stable platelet counts over extended periods.	Potential risk of severe bleeding.
	2	Personalized dosing strategy increases safety and efficacy.	After treatment cessation there is increased risk of bone marrow fibrosis and thromboembolic episodes.
	3	Possibility of a decrease/termination of steroids.	Common TEAEs: dizziness + joint pain.
	4	Suitable for those with chronic ITP who are more vulnerable to hemorrhage.	Enhanced risk of hematological malignancies and myelodysplastic syndromes.
	5	Safe, effective, and well tolerated in pediatric patients.	Uncomfortable and potential of injection site infection.

### 3.4. Adverse Events (AEs) for Each ITP Treatment

**Figure 6:**
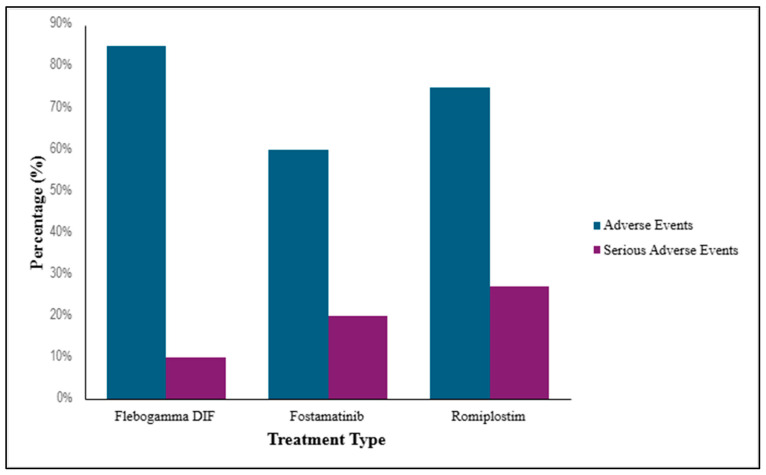
The average percentage of AEs and SAEs associated with each treatment.

The FDA describes AEs and SAEs as any unfavorable experiences related to the use of a medical product in a patient [68]. Figure 6 shows that Flebogamma DIF is associated with the highest proportion of mild-to-moderate AEs, while SAEs remain uncommon. Fostamatinib commonly produced gastrointestinal symptoms and hypertension, with moderate rates of AEs. Romiplostim demonstrated the highest proportion of SAEs, including severe bleeding. This graph demonstrates how quickly each treatment can increase and maintain the platelet response [69].

### 3.5. Platelet Response and Sustained Platelet Response per Treatment in Percentage (%)

**Figure 7:**
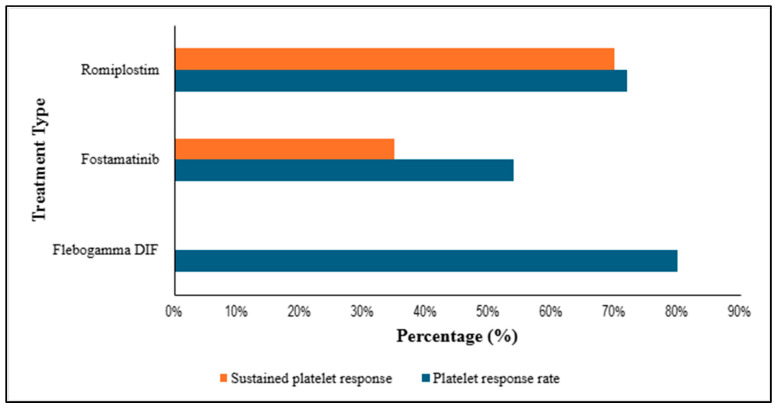
An estimate of the overall platelet response vs. sustained platelet response between the different ITP treatments.

Flebogamma DIF was reported to show the highest initial response but no sustained response. Romiplostim was reported to demonstrate both high initial and high sustained responses. Fostamatinib was reported to exhibit moderate initial responses and lower sustained responses relative to Romiplostim. These patterns reflect differences in mechanism of action and intended treatment duration. Figure 7 summarizes overall and sustained platelet responses [70].

### 3.6. The Mean Time to Response in Days for Each Treatment Option

**Figure 8:**
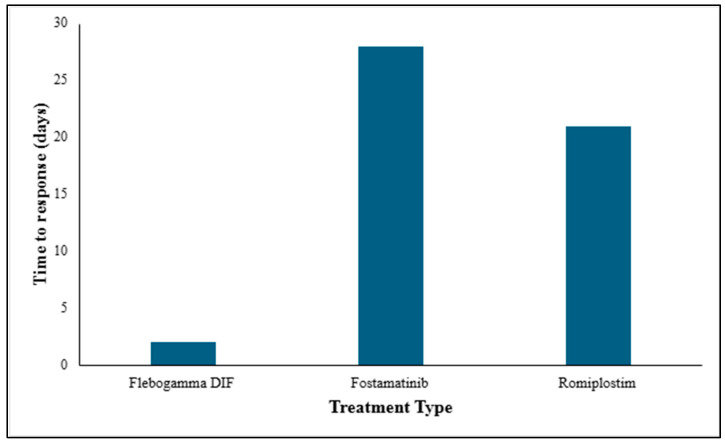
The average time taken in days for treatment to generate a significant increase in platelet count.

Flebogamma DIF provides the fastest response (~2 days), followed by Romiplostim (~21 days) and Fostamatinib (~28 days). The route of administration of a drug influences the rate at which a response is produced (Figure 8). Oral drugs are required to move through the gastrointestinal (GI) system, exposing it to intestinal absorption and hepatic first-pass metabolism [71]. Parenteral administration provides increased drug response rates, compared with other routes.

Overall, most studies displayed a moderate risk of bias, mainly because of limitations in outcome characteristics commonly associated with non-randomized designs. Several studies were judged to have a serious risk of bias, largely driven by small sample sizes, heterogeneous patient populations, and lack of blinding. Missing data and selective reporting were generally well-addressed across studies. A detailed breakdown of domain-level assessments for each study is provided in Supplementary Material (Tables S1–S3).

## 4. Discussion

Comparative interpretations in this review are limited by heterogeneity in study design and variable risk of bias. Evident differences in efficacy, such as higher response rates reported for Romiplostim, represent outcomes across individual studies rather than a combined estimate. These limitations reinforce that ITP treatment should be guided by individual patient-specific factors such as disease duration, comorbidities, and treatment access, highlighting the importance of an individualized treatment approach.

### 4.1. Comparative Analysis of Efficacy and Safety Profiles: Key Findings

Flebogamma DIF was reported to demonstrate the fastest-onset action, achieving the desired platelet count in around 2 days, supporting its use in situations requiring rapid platelet increase [72]. Its effect is short-lived and often requires repeated infusions, which may increase toxicity and cost [73]. Although AEs are common, most are mild and self-limiting [74]. Combination therapy with IV methylprednisolone has demonstrated the ability to increase the platelet count more rapidly; however, evidence remains limited and further research is needed to confirm its role in critical settings [18].

Romiplostim produced platelet responses in around 21 days while Fostamatinib had the slowest onset at around 28 days. The delayed response may be due to its oral administration, which is subject to GI absorption and first-pass metabolism, unlike parenteral therapies [75,76].

Fostamatinib may offer advantages over Flebogamma DIF for longer-term platelet stabilization, although its sustained response remains moderate. Studies have shown that outcomes are better when used earlier in the treatment pathway, with higher response rates and fewer bleeding episodes in second-line settings [77]. These findings raise the question of whether Fostamatinib could have a role earlier in the treatment pathway, although evidence remains insufficient to support its first-line use. It is associated with GI issues and hypertension, but unlike Romiplostim, it is associated with fewer thrombotic episodes, providing a potential viable alternative for patients at higher risk of thrombosis [4]. Most AEs are mild to moderate.

In many studies, Romiplostim has demonstrated the highest sustained platelet responses, supporting its role in chronic ITP and in patients unresponsive to previous therapies [78]. However, it carries increased risks of thrombosis and liver damage; therefore, it may not be suitable for patients who have previously experienced thromboembolism, who are older or obese, or who are currently taking estrogen or progesterone treatment types [79,80]. Although it can induce treatment-free remission, it also has the highest proportion of SAEs, highlighting the need to balance efficacy with safety considerations [81].

Overall, Romiplostim may be preferred in cases requiring rapid and sustained platelet recovery, while Fostamatinib may be a better alternative for patients who cannot access or tolerate parenteral therapies; nevertheless, treatment choice should remain individualized. Flebogamma DIF remains widely used for acute management, particularly in pediatric and emergency settings. These findings suggest that each treatment has a distinct impact on platelet counts, with suitability depending on whether the therapeutic goal is short-term or long-term management, as well as patient-specific factors such as age, comorbidities, and disease duration [82]. Since no head-to-head trials exist, claims regarding their superior efficacy are based on indirect comparisons across heterogeneous studies which vary in patient populations, study design, sample size, therapeutic outcomes, and ITP classification; therefore, all comparative interpretations should be viewed cautiously.

### 4.2. Integration into Clinical Practice

Integrating these therapies into clinical practice requires consideration of disease stage, urgency, comorbidities, and prior treatment response. IVIG, such as Flebogamma DIF is typically used for acute, short-term platelet elevation, particularly when a rapid platelet increase is crucial. When ITP becomes persistent or chronic, TPO-Ras such as Romiplostim are commonly used to maintain sustained platelet production, which is consistent with ASH and international guideline recommendations. When it comes to tackling refractory ITP, Syk inhibition therapies such as Fostamatinib may be considered for adults who have failed to respond to first-line therapy, although it is not approved for pediatric use due to its effects on bone development, as noted in current guideline recommendations. Switching between ITP treatments based on mechanism of action, safety and patient-specific factors is common in practice. Evidence for combination therapy remains limited due to insufficient case reports.

### 4.3. Treatment Failure and Long-Term Safety Considerations

The major challenges surrounding ITP treatment are the inconsistency in patients’ response to various treatments [83,84]. Treatment responses in ITP remain highly variable, and each therapy carries distinct risks of failure or relapse. Flebogamma DIF provides short-term benefits, which often leads to relapses and the need for repeated infusions, which may increase infection risk and reduce long-term feasibility [27]. Studies have reported that there are higher discontinuation rates for Fostamatinib due to persistent AEs, while Romiplostim tends to have a lower discontinuation rate, while sudden treatment discontinuation has been associated with rebound thrombocytopenia [54,85]. Regular monitoring is essential to ensure safe long-term use.

Each therapy carries distinct long-term safety risks that must be balanced against therapeutic benefit [86]. Repeated high doses of Flebogamma DIF can result in serious renal dysfunction, thrombosis, arrhythmia, aseptic meningitis, etc. [87]. Given that Fostamatinib is a spleen tyrosine kinase inhibitor (TKI), it can cause dangerous hypertension, liver toxicity, febrile neutrophilia, and pneumonia [88,89]. Since Romiplostim operates in platelet stimulation, over extended periods of time this can result in severe complications such as enhanced bone reticulin marrow, especially in patients with cardiovascular issues [90]. These considerations highlight the importance of tailoring treatments based on individualized treatment factors and therapeutic goals.

### 4.4. Clinical Relevance of ITP Treatments

Each treatment operates efficiently for various patient subgroups depending on their therapeutic requirements and risks [35]. Treatment choices are based on clinical urgency and patient-specific factors. Flebogamma DIF is commonly used when rapid platelet elevation is required [91]. Fostamatinib and Romiplostim are more suitable for sustained platelet stabilization, while Romiplostim has shown durable responses in several studies, though direct comparisons between the two are lacking. Sequential or combination therapy has been explored as a potential approach in refractory or complex cases, but evidence remains limited to determine its benefit. Combining Flebogamma DIF and Romiplostim may be beneficial in severe refractory ITP due to their complementary mechanisms. Fostamatinib’s oral administration may improve patient compliance, but its use is restricted in pediatric patients due to safety concerns.

### 4.5. Treatment Analysis

Flebogamma DIF, Fostamatinib and Romiplostim have different mechanisms of action which influence the short- and long-term platelet responses in ITP. Although the mechanism through which Flebogamma DIF operates is not fully comprehended, it involves immunomodulatory effects which rapidly increase platelet counts by inhibiting Fc-mediated platelet destruction, supporting its use acute in ITP [27,42]. Its short duration and need for repeated infusions may increase the likelihood of adverse events and can potentially lead to drug tolerance, making it less effective for chronic ITP that requires long-term treatment [92,93].

Romiplostim binds to the thrombopoietin receptor on hematopoietic cells in bone marrow, which stimulates various signaling pathways, resulting in an increase in platelet production [94]. This mechanism allows Romiplostim to provide sustained platelet responses, particularly in chronic ITP patients who require long-term, ongoing platelet management [95]. Additionally, recent studies suggest that Romiplostim may also possess immune-regulating effects, which have been hypothesized to contribute to treatment-free sustained response, but the extent of this effect is still not fully comprehended and further research will be required to validate this effect [96]. However, as Romiplostim does not address the root cause of the autoimmune platelet destruction, this indicates that platelets could potentially undergo destruction at a rate greater than platelet production or, conversely, platelet production can become excessive. This imbalance and unpredictability can result in many complications [97].

Fostamatinib inhibits the Syk signaling pathway, reducing immune-mediated platelet destruction and offering a targeted option for patients who have failed to respond to previous treatments [63,98]. These mechanistic differences highlight the importance of individualized treatment planning.

### 4.6. Global Distribution of ITP Treatment

Global access to ITP therapies varies widely due to cost and infrastructure. In 2018 the global ITP market was predicted to be valued at approximately $3.30 billion by 2032, where North America is said to have a market share of 52.84%, dominating the market [99]. However, higher treatment intensity has been reported in some high-income regions, with ITP treatment estimated to be around $4700 per person [100]. In low-resource settings, access is limited by manufacturing costs and pricing. Since Flebogamma DIF involves costly manufacturing and production processes, this has led to challenges in the availability of this treatment worldwide [101]. Similarly, as Romiplostim is an expensive therapy with a 6-month treatment period estimated to cost around $34,655 [83], this has led to the development of a biosimilar called Romy, which was produced and introduced in India with a monthly cost of $160 and has provided an inexpensive treatment alternative to Romiplostim in resource-limited settings [102]. Fostamatinib, being an oral therapy, may reduce healthcare burden by avoiding infusion-based administration, although its initial cost and inconsistent insurance coverage can still limit accessibility [103,104]. Addressing these limitations is essential to improve global access to effective ITP treatments.

### 4.7. Gaps in the Literature and Limitations

While substantial advancements have been achieved in ITP treatment, many gaps persist. Further research is needed to refine treatment strategies, improve outcomes, and enhance global accessibility. A significant limitation of this review is the lack of clear and consistent classification of ITP types across existing studies. While international guidelines differentiate ITP into acute, persistent, and chronic, many studies fail to explicitly state the disease duration or subtype. Correspondingly, the immune vs. non-immune categorization was absent, limiting the analysis. Therefore, this review mainly addresses immune ITP, and comparisons across ITP types remain limited. As a result, this review primarily reflects findings related to immune ITP, and comparisons across ITP categories remain limited.

Another significant gap is the lack of direct comparative studies. Most available evidence evaluates a single therapy in isolation rather than comparing two or more treatments within the same trial, making it difficult to identify which therapy may be superior in terms of efficacy for different populations. Indirect comparisons are unavoidable but are subject to bias due to variations in patient attributes across trials [34]. The absence of head-to-head trials limits the ability to determine true comparative efficacy, durability, cost-effectiveness, and tolerability across patient populations.

While a personalized approach or combination therapy is emphasized to maximize ITP treatment, the lack of robust research around their long-term effectiveness and patient-specific responses limits their applications. To optimize the development of a personalized approach in ITP management, a deeper comprehension and analysis of genetic, immunological, and clinical elements is essential to evaluate the impact of Flebogamma DIF, Fostamatinib and Romiplostim on patient-specific factors [43]. Similarly, although combination therapy may theoretically provide both rapid and sustained platelet responses, definitive evidence regarding its safety and benefit is insufficient, confirming the need for more research on these treatment options.

As a narrative systematic review, this study is limited by heterogeneity in study methodologies, outcome definitions, and reporting quality. The lack of quantitative synthesis such as meta-analysis reflects the variability across studies, including the absence of statistical measures such as hazard ratios, confidence intervals, or Kaplan–Meier survival analyses. These factors limit the strength of comparative conclusions, highlighting that observed differences may reflect study-level heterogeneity rather than true treatment effects. Therefore, publication bias and selective reporting cannot be excluded, particularly for newer therapies with limited long-term data.

Addressing these gaps through standardized reporting, well-designed comparative trials, and comprehensive long-term studies will be essential to advance ITP management, enabling healthcare professionals to make knowledgeable decisions regarding patient-specific treatment approaches, optimizing patient outcomes. The overall strength of the evidence is limited by the greater proportion of non-randomized studies, many of which demonstrated moderate to serious risk of bias due to confounding, small sample sizes, and unblinded outcome assessment. These methodological limitations should be considered when interpreting comparative findings across treatments.

## 5. Conclusions

This research provides a comprehensive analysis of Flebogamma DIF, Fostamatinib and Romiplostim in the management of ITP for both adult and pediatric patients. The review compared their durability, safety profile, mechanism of action, and global availability. Each therapy operates in a distinct manner in ITP management. Flebogamma DIF and Fostamatinib primarily reduce immune-mediated platelet destruction; however, Flebogamma DIF does so via immune modulation, while Fostamatinib inhibits the Syk pathway.

Romiplostim has demonstrated sustained platelet responses in multiple clinical studies, although its use must be balanced against its adverse event profile and higher treatment costs. Flebogamma DIF remains a viable option when a rapid platelet response is required, especially in emergency situations, due to its ability to increase platelet counts within 24–48 h. Nonetheless, its short-term and transient effect, along with high costs, limits its applicability, especially in low-income regions. Fostamatinib offers an oral alternative that may be suitable for some patients with refractory ITP and for those in resource-limited settings, though its side-effect profile and variable response rates require careful consideration. These findings underscore the importance of personalized treatment strategies that consider patient-specific factors such as disease severity, treatment history, comorbidities, and healthcare infrastructure. No single therapy is universally effective, highlighting the need for tailored approaches in ITP management.

In comparison with existing literature on ITP treatment, this study provides a focused evaluation of three targeted therapies with distinct mechanisms of action, illustrating how these mechanisms impact their efficacy and safety profiles. This review highlights the need for further long-term, comparative studies to guide evidence-based ITP management. Therefore, by addressing economic and accessibility challenges in ITP treatment, this review emphasizes the ongoing need for affordable, accessible treatment options to support efficient ITP care worldwide.

## Data Availability

The data presented in this study were obtained from previously published journal articles available in the public domain. The data supporting the findings of this study are already cited within the manuscript.

## Supplementary Materials

The following supporting information can be downloaded at https://www.mdpi.com/article/10.3390/life16030440/s1: Table S1. Risk-of-bias assessment for Flebogamma DIF studies using the ROBINS-I tool; Table S2. Risk-of-bias assessment for Fostamatinib studies using the ROBINS-I tool; Table S3. Risk-of-bias assessment for Romiplostim studies using the ROBINS-I tool.

## Abbreviations

ITP	Immune thrombocytopenia
DIF	Intravenous immunoglobulin
PRISMA	Preferred Reporting Items for Systematic Reviews and Meta-Analyses
Syk	Spleen tyrosine kinase
IVIG	Intravenous immunoglobulin
TPO-RA	Thrombopoietin receptor agonists
IV	Intravenous
SC	subcutaneous
AE	Adverse event
SAE	Serious adverse event
GI	Gastrointestinal
FDA	Food and Drug Administration
T-cell	T lymphocyte
B-cel	B lymphocyte
EMA	European Medicines Agency
JAK	Janus Kinase
STAT	Signal transducer and activator of transcription
MAPK	Mitogen-activated protein kinase P42/P44, 42/44 kDa
SHC, Src	Homology 2 domain-containing
GRB2	Growth factor receptor-bound protein 2
SAS	Src adapter proteins
RAF	Rapidly accelerated fibrosarcoma
RAS	Rat sarcoma
TEAE	Treatment-emergent adverse event
PRR	Platelet response rate
SR	Stable response
ORR	Overall response rate
CR	Complete response
IBS	Irritable bowel syndrome
RoB	Risk of bias
