# Supplementary material for: A Comparative Analysis of the Efficacy, Safety and Mechanism of Action of Flebogamma DIF, Fostamatinib and Romiplostim in Immune Thrombocytopenia
Source: Life (Basel). 2026 Mar 9;16(3):440. doi: 10.3390/life16030440 (PMC13027866; doi:10.3390/life16030440)
Supplement: Supplementary file 1 [file life-16-00440-s001.zip › life-4114625-supplementary.pdf]

SUPPLEMENTARY MATERIAL

A Comparative Analysis of the Efficacy, Safety and Mechanism of Action of Flebogamma DIF, Fostamatinib and Romiplostim in Immune Thrombocytopenia

Mary Akinyemi[1,2], Kamna Ravi[3], Furong Tian[1,2,*] and Baljit Singh [2,3]

1 School of Food Science and Environmental Health, College of Sciences and Health, Technological University Dublin (TU Dublin), D07 H6K8 Dublin, Ireland.
2 NanoLab Research Centre, Physical to Life Sciences Research Hub, Technological University Dublin (TU Dublin), D08 CKP1 Dublin, Ireland.
3 MiCRA Biodiagnostics Technology Gateway and Health, Engineering & Materials Science (HEMS) Research Hub, Technological University Dublin (TU Dublin), D24 FKT9 Dublin, Ireland.

* Correspondance: furong.tian@tudublin.ie (F.T.)

Risk-of-Bias Assessment

This section provides methodological details that support the interpretation of the main findings used in this narrative review. This section includes the full risk-of-bias assessment for all included studies, evaluated using the ROBINS-I tool. The data below offers transparency regarding potential confounding, selection processes, outcome measurement limitations, and the overall study quality. By presenting these details in the appendix ensures clarity without interrupting the flow of the main text. This assessment is crucial for understanding the strengths and limitations of this study.

Table S1. Risk-of-bias assessment for Flebogamma DIF studies using the ROBINS-I tool.

Study (Ref)	Confounding	Selection	Classification of Intervention	Deviations	Missing Data	Outcome Measure	Reported Results	Overall ROB
Study 1: Apte et al. (2019)	Moderate	Low	Low	Low	Low	Moderate	Low	Moderate
Study 2: Kovaleva et al. (2016)	Moderate	Low	Low	Low	Low	Moderate	Low	Moderate
Study 3: Alsina et al. (2017)	Serious	Moderate	Low	Low	Low	Moderate	Low	Serious
Study 4: Julia (2011)	Moderate	Low	Low	Low	Low	Moderate	Low	Moderate
Study 5: Varga et al. (2006)	Moderate	Low	Low	Low	Low	Moderate	Low	Moderate

Table S2. Risk-of-bias assessment for Fostamatinib studies using the ROBINS-I tool.

Study (Ref)	Confounding	Selection	Classification of Intervention	Deviations	Missing Data	Outcome Measure	Reported Results	Overall ROB
Study 1:	Moderate	Low	Low	Low	Low	Moderate	Low	Moderate

SUPPLEMENTARY MATERIAL

Berliner and Connell (2019)								
Study 2: Kuwana et al. (2023)	Moderate	Low	Low	Low	Low	Moderate	Low	Moderate
Study 3: Gonzalez-Lopez et al. (2024)	Serious	Moderate	Low	Low	Low	Moderate	Low	Serious
Study 4: Gonzalez-Lopez et al. (2023)	Serious	Moderate	Low	Low	Low	Moderate	Low	Serious
Study 5: Boccia et al. (2019)	Moderate	Low	Low	Low	Low	Moderate	Low	Moderate
Study 6: Jiménez-Bárcenas et al. (2024)	Moderate	Low	Low	Low	Low	Moderate	Low	Moderate
Study 7: Lee, Izak and Bussel (2020)	Serious	Moderate	Low	Low	Low	Moderate	Low	Serious

Table S3. Risk-of-bias assessment for Romiplostim studies using the ROBINS-I tool.

Study (Ref)	Confounding	Selection	Classification of Intervention	Deviations	Missing Data	Outcome Measure	Reported Results	Overall ROB
Study 1: Obara et al. (2024)	Serious	Moderate	Low	Low	Low	Moderate	Low	Serious
Study 2: Zhou et al. (2023)	Moderate	Low	Low	Low	Low	Moderate	Low	Moderate
Study 3: Christiansen et al. (2024)	Moderate	Moderate	Low	Low	Low	Moderate	Low	Serious
Study 4: Snell et al. (2021)	Moderate	Low	Low	Low	Low	Moderate	Low	Moderate
Study 5: Tarantino et al. (2020)	Moderate	Low	Low	Low	Low	Moderate	Low	Moderate
Study 6: Schifferli et al. (2023)	Moderate	Low	Low	Low	Low	Moderate	Low	Moderate
Study 7: Grainger et al. (2023)	Serious	Moderate	Low	Low	Low	Moderate	Low	Serious
Study 8: Chandana Mareddy, Kalra and Sachdeva (2022)	Moderate	Low	Low	Low	Low	Moderate	Low	Moderate
